# Determinants of conventional and contrast-enhanced ultrasound diagnosis of fat-poor angiomyolipoma <5 cm

**DOI:** 10.3389/fonc.2024.1446801

**Published:** 2024-12-13

**Authors:** Xia Liang, Xian-Tao Zeng, Zhi-Liang Hong, Miao-Jiao Su, Jian-Chuan Yang, Song-Song Wu

**Affiliations:** ^1^ Fujian Provincial Hospital, Provincial Clinical Medical College, Fujian Medical University, Fuzhou, China; ^2^ Fujian Provincial Hospital, Fujian Provincial Key Laboratory of Critical Care Medicine, Fuzhou, China; ^3^ Department of Ultrasound, Fujian Provincial Hospital, Fuzhou, China

**Keywords:** fat-poor angiomyolipoma, renal cell carcinoma, contrast-enhanced ultrasound, conventional ultrasound, starry-sky sign

## Abstract

**Purpose:**

This study aims to assess the diagnostic efficacy of conventional ultrasound (CUS) and contrast-enhanced ultrasound (CEUS) in detecting fat-poor angiomyolipomas(AML) with dimensions less than 5 cm. Additionally, the study seeks to identify independent indicators for predicting the presence of fat-poor AML.

**Methods:**

We conducted a retrospective analysis of patients diagnosed with renal AML and renal cell carcinoma, who were admitted and underwent surgery at Fujian Provincial Hospital from January 2013 to October 2023. A total of 154 renal tumors were included (104 renal cell carcinomas and 50 fat-poor AMLs). Prior to radical or partial nephrectomy, these patients underwent both CUS and CEUS examinations. We systematically analyzed the features observed in CUS and CEUS, identified independent factors through multifactorial regression analysis, and evaluated diagnostic efficacy by calculating the area under the curve (AUC).

**Results:**

Univariate analysis revealed significant distinctions in fat-poor AML concerning gender, age, morphology, internal hyperechoic features (starry-sky sign, crescent sign), enhancement uniformity, and delayed enhancement, all displaying significance compared to renal cell carcinoma (RCC) (*p* < 0.05). Multivariate analysis demonstrated that internal hyperechoic features (*p* < 0.01, Odds Ratio [OR] = 0.003, 95% Confidence Interval [CI]: 0.000-0.0028) and enhancement uniformity (*p* < 0.01, OR = 0.016, 95% CI: 0.001-0.229) independently predicted fat-poor AML. The Receiver Operating Characteristic (ROC) curve’s area under the curve (AUC) for internal hyperechoic features (starry-sky sign, crescent sign) was 0.88 (95% CI: 0.80–0.95), with a sensitivity of 78.00%, specificity of 97.12%, positive predictive value of 92.85%, and negative predictive value of 90.18%. Conversely, the ROC curve AUC for enhancement uniformity was 0.70 (95% CI: 0.62–0.78), with a sensitivity of 96.00%, specificity of 44.23%, positive predictive value of 45.28%, and negative predictive value of 95.83%.

**Conclusion:**

This study suggests that both CUS and CEUS possess discriminative value in differentiating fat-poor AMLs from RCCs. Notably, internal hyperechoic features (starry-sky sign, crescent sign) and uniform enhancement within renal tumors emerge as potential independent indicators for predicting fat-poor AML.

## Introduction

Renal angiomyolipoma (AML), characterized by the presence of smooth muscle, blood vessels, and adipose tissue, stands as the most prevalent benign tumor within the kidney (1, 2) and is affiliated with the PEComa family, constituting 2.0-6.4% of all renal tumors. Most AML cases require only follow-up observation without the need for surgical intervention (3). Among them, epithelioid AML is characterized by perivascular epithelioid cells. Although it has malignant potential, partial nephrectomy, such as robot-assisted partial nephrectomy (4), and minimally invasive treatments (e.g., radiofrequency ablation, microwave ablation) are options (5). However, smaller lesions can be managed conservatively, similar to traditional AML treatment (6, 7). In contrast, renal cell carcinoma (RCC) is treated primarily through surgical resection (8, 9). However, the challenge arises in distinguishing fat-poor AMLs from RCC, which may lead to misdiagnosis. Reports indicate that for renal tumors smaller than 4 cm, partial nephrectomy often results in a benign pathology, with half of these cases being AML (10, 11). Hence, achieving a clear distinction between fat-poor AMLs and RCC in imaging is imperative. This distinction aids in determining whether essential surgery or vigilant follow-up is warranted (12).

In most instances, the typical ultrasound (US) presentation of renal AML is hyperechoic, facilitating a clear distinction from malignant tumors and establishing itself as a highly specific marker for AML (13–15). Fat-poor AML can be classified into hyperattenuating type and isoattenuating type based on the findings of unenhanced CT (14), with hyperattenuating type accounting for 4-5% of AML (16). Pathologically, the fat content of hyperattenuation type is less than 4%, while that of the isoattenuating type pathologically has less than 25% fat content (14, 15). To date, careful interpretation of computed tomography (CT) and magnetic resonance imaging (MRI) have certain advantages in differentiating fat poor AML from RCC (14, 16). However, it often remains challenging and requires further renal mass biopsy (14, 16–19), which may introduce additional risks, costs, and potential contraindications for certain patients (20). With continuous advancements in US technology, two-dimensional US now offers greater clarity. The use of color Doppler US, three-dimensional US, and particularly the discovery of contrast-enhanced ultrasound(CEUS), has become increasingly valuable. Although there has been several researches on the combination of conventional US and CEUS for differentiating classical AMLs from RCC, limited research exists on their effectiveness in distinguishing fat-poor AMLs from RCC (3). Our objective is to pinpoint distinctive imaging features on ultrasound and CEUS, mitigating the risk of misdiagnosing fat-poor AMLs, thereby reducing unnecessary surgical interventions and benefiting patients. Recognizing that larger renal tumors may pose challenges due to hemorrhage, necrosis, cystic changes, or metastasis interfering with imaging interpretation, our study concentrates on tumors smaller than 5 cm.

This study aims to assess the diagnostic effectiveness of CUS and CEUS in discriminating fat-poor AMLs from RCC, and to identify independent indicators predicting fat-poor AMLs.

## Materials and methods

### Patients

This study is a single-center retrospective analysis. The study was approved by the Institutional Review Board of Fujian Provincial Hospital, and written informed consent was obtained from all patients, who were aware of and consented to undergoing contrast-enhanced ultrasound examinations. A total of 57 patients with fat-poor AMLs underwent partial or radical nephrectomy between January 2013 and October 2023. Additionally, 114 RCC patients who underwent partial or radical nephrectomy during the same period were randomly selected in a 1:2 ratio. Inclusion criteria for the study were as follows: patients underwent CUS and CEUS before radical or partial nephrectomy; lesions exhibited hypoechoic or predominantly hypoechoic characteristics; solid masses with a maximum diameter less than 5 cm; and patients had not undergone any invasive treatment before CUS and CEUS. The study enrolled a total of 156 patients with 159 renal tumors, consisting of 53 cases with fat-poor AMLs and 106 cases of RCC. Exclusion criteria for CUS and CEUS analysis included: unclear CUS images; predominantly cystic lesions (n=1); lack of contrast enhancement (n=2), or inadequate contrast enhancement due to a brief recording time (n=1) or image jitter (n=1).

Ultimately, this study included 154 cases, comprising 50 fat-poor AMLs, with 20 males and 30 females, and a mean age of 48.54 ± 12.71 years. There were 104 RCC cases, with 64 males and 38 females, and a mean age of 53.46 ± 15.43 years.

### CUS and CEUS examination

CUS and CEUS examinations were performed by a single ultrasound specialist (W.S.S and C.S), possessing 20 years of abdominal ultrasound experience and 13 years of specialized expertise in CEUS at our institution. Utilizing a Philips iU22 color Doppler ultrasound system equipped with a C5-2 transducer (frequency 2-5 MHz) for the initial CUS assessment, the objective was to ascertain the tumor’s location, size, morphology, and internal echoes. Subsequently, the most optimal section covering the entire renal lesion and adjacent normal renal parenchyma was chosen, prompting a transition to CEUS mode. In this study, we employed SonoVue (Bracco, Milan, Italy) as the contrast agent, comprised of sulfur hexafluoride microbubbles stabilized by phospholipids. The SonoVue lyophilized powder was reconstituted with 5.0 ml of normal saline to form a suspension. Depending on the patient’s weight, height, and age, 1.6-2.2 ml of the suspension was administered through the antecubital vein, followed by a 5.0 ml saline flush. The injection was precisely synchronized with the timer and video recorder buttons. Patients maintained a slow breathing pattern, and each dynamic contrast-enhanced image was observed for a minimum of 3 minutes. If the assessment of the tumor yields suboptimal results, a second injection is administered 15 minutes after the initial administration. Individual images and video clips from both CEUS and CUS are meticulously stored on a local hard drive for subsequent analysis.

### Imaging interpretation and data evaluation

The images and video clips stored on the local hard drive underwent independent, blinded review by two ultrasound physicians (L.X. and Z.X.T), with a random allocation, ensuring no knowledge of the pathological outcomes. Both ultrasound physicians, each boasting over 7 years of expertise in abdominal ultrasound interpretation and 5 years in CEUS reading, remained uninformed about the pathological results. Assessment of the CUS images encompassed tumor location, size, shape, and internal hyperechoic features. Internal hyperechoic features within the mass were defined as echoes resembling those of the renal sinus, presenting as either punctate (starfield sign) or arc-shaped (crescent sign). Echogenic features not slightly exceeding the renal sinus echo were excluded, as were strong echoes surpassing the renal sinus echo, accompanied by a comet tail artifact.

With reference to the normal renal cortex adjacent to the renal mass, we conducted an analysis of the CEUS features of the renal mass. The CEUS characteristics encompassed intensity level, enhancement uniformity, enhancement pattern, and washout pattern. Intensity levels were categorized as high enhancement, iso-enhancement, and low enhancement. Enhancement uniformity was classified as either uniform or non-uniform. Uniformity indicated a singular form of enhancement pattern, while non-uniformity suggested a diverse form of enhancement. Enhancement patterns included rapid-in, synchronous, and slow-in, while washout patterns encompassed rapid-out, synchronous, and slow-out. In instances where there was a disparity in conclusions between the two ultrasound physicians, a consultation with a third, more experienced ultrasound specialist was sought to achieve a final consensus through thorough discussion.

### Statistical analysis

The quantitative data were presented as mean ± standard deviation (SD). Independent sample t-tests and Mann-Whitney U tests were applied for comparing quantitative data between fat-poor AML and RCC. Pearson’s chi-square test and Fisher’s exact test were employed for comparing categorical data between groups. Multivariate logistic regression analysis was conducted for variables that exhibited favorable performance in univariate analysis, aiming to predict fat-poor AML. Odds ratios (OR), 95% confidence intervals (CIs), and p-values were calculated. Variables demonstrating significant and independent effects were isolated to construct Receiver Operating Characteristic (ROC) curves, determining the area under the ROC curve (AUC). The optimal cutoff value for the representative index with the highest AUC was identified. Sensitivity, specificity, positive predictive value, and negative predictive value were computed accordingly. Statistical analysis was performed using IBM SPSS Statistics version 22.0 (IBM Corporation, Armonk, NY, USA). A *p*-value < 0.05 was considered statistically significant.

## Results

### Characteristics of the enrolled patients

A total of 154 cases of renal tumors were enrolled in this study, with 104 cases (67.53%) identified as RCCs and 50 cases (32.47%) as fat-poor AMLs. Within the RCCs subgroup, clear cell carcinomas (ccRCCs) comprised 93 cases (89.42%), papillary renal cell carcinomas (pRCCs) accounted for 6 cases (5.77%), and chromophobe renal cell carcinomas (cRCCs) were present in 5 cases (4.81%). Pathologically, all AML lesions demonstrated ≤15% fat content, consistent with the diagnosis of fat-poor AML. Eight cases exhibited an epithelioid AML subtype, while 42 cases were classified as classical AMLs, showcasing a predominant presence of epithelioid and spindle cells under microscopic examination. Significantly divergent age and gender distributions were noted between patients with fat-poor AMLs and RCCs (*p*<0.05) (**Table 1**).

**Table 1:** Patient clinical characteristics.

Characteristics	Description	fat-poor AML	RCC	x ^2^/t	*p*-value
Gender	Male	20	68	8.89	<0.01
	Female	30	36		
Age	mean ± SD (years)	48.54± 12.71	53.46 ± 15.43	2.10	0.04
Laterality	Left kidney	33	59	1.21	0.27
	Right kidney	17	45		
Tumor location	Upper pole	16	31	0.60	0.74
	Middle part	17	42		
	Lower pole	17	31		

### CUS characteristics of renal masses

**Figure 1:**
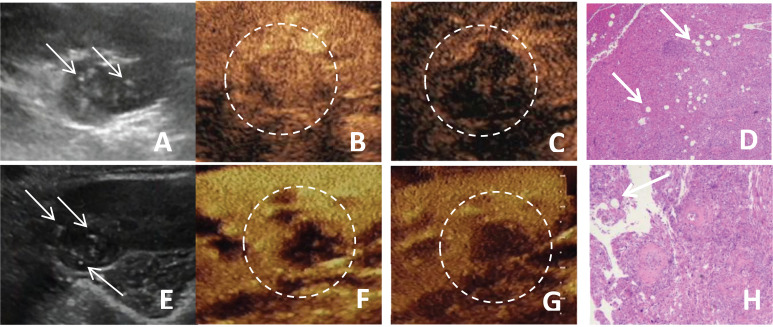
**(A-D)** A 51-year-old female patient with a fat-poor angiomyolipoma(AML) in the left kidney; **(E-H)** A 44-year-old female patient with a fat-poor AML in the right kidney. **(A, E)** Ultrasound images of fat-poor AML demonstrate heterogeneous internal echoes with multiple foci of hyperechoic regions (indicated by arrows). **(B)** Contrast-enhanced ultrasound (CEUS) shows a homogeneously iso-enhanced peak. **(C)** CEUS shows low enhancement in the late phase. **(F)** CEUS shows a homogeneously low-enhanced peak. **(G)** CEUS shows low enhancement in the late phase. **(D, H)** Corresponding histopathological sections show scattered clusters of adipocytes (indicated by arrows) (original magnification, ×10; hematoxylin and eosin [H&E] staining).

**Figure 2:**
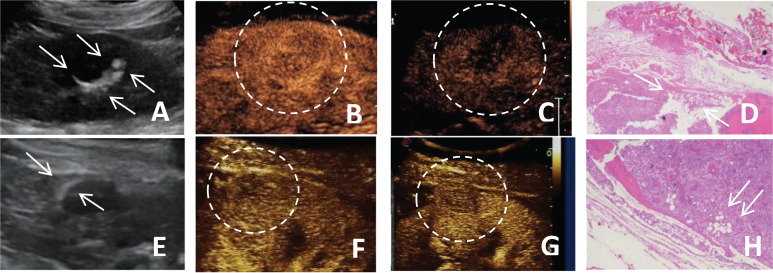
**(A-D)** A 51-year-old female patient with a fat-poor angiomyolipoma(AML) in the left kidney; **(E-H)** A 48-year-old female patient with a fat-poor AML in the right kidney. **(A, E)** Ultrasound images of fat-poor AML show crescent-shaped hyperechoic areas along the lesion margins (indicated by arrows). **(B)** Contrast-enhanced ultrasound (CEUS) shows a homogeneously hyper-enhanced peak. **(C)** CEUS shows low enhancement in the late phase. **(F)** CEUS shows a homogeneously iso-enhanced peak. **(G)** CEUS shows low enhancement in the late phase. **(D, H)** Corresponding histopathological sections show scattered clusters of adipocytes (indicated by arrows) (original magnification, ×10; hematoxylin and eosin [H&E] staining).

Fat-poor AML demonstrate noteworthy distinctions from RCC concerning internal hyperechoic features, yielding a *p*-value of < 0.01. Among fat-poor AMLs, 39 out of 50 cases exhibit internal hyperechoic signals, characterized by either a punctate distribution (resembling a starry sky) or a linear/arcuate distribution (resembling a crescent moon) (**Figures 1**, **2**). In contrast, only 3 out of 104 RCC cases display internal hyperechoic signals, primarily with a punctate distribution. Both fat-poor AML and RCC frequently manifest with regular shapes, predominantly circular or oval. Nevertheless, irregular shapes are noted in 7 out of 50 fat-poor AML cases and 3 out of 104 RCC cases, with the mushroom-like configuration being a prevalent variation (**Table 2**). No statistically significant distinctions were discerned concerning tumor position, location, or size between fat-poor AMLs and RCCs.

**Table 2:** CUS characteristics of renal masses.

Characteristics	Description	fat-poor AML	RCC	*x ^2^/t*	*p*-value
Size	mean ± SD (mm)	2.74± 1.11	2.75± 1.17	0.07	0.95
Shape	Round/Oval	43	101	5.16	0.02
	Irregular	7	3		
Echogenicity	Hyper-echoic	0	0	86.55	*<*0.01
	Iso-echoic	0	83		
	Hypo-echoic	50	21		
hyperechoic	exist	39	3	96.05	*<*0.01
	inexist	11	101		

### CEUS characteristics of renal masses

The CEUS characteristics of renal masses are summarized in **Table 3**. Noteworthy distinctions exist between fat-poor AMLs and RCCs regarding enhancement uniformity and delayed washout, both demonstrating significant differences (both *p* values < 0.00). However, there were no statistically significant variations observed in peak intensity, rapid inflow, plateau phase, slow inflow, rapid outflow, or plateau phase outflow.

**Table 3:** CEUS characteristics of renal masses.

Characteristics	Description	fat-poor AML	RCC	x ^2^/t	*p*-value
Enhancement intensity	Hyper-enhancement	35	57	3.80	0.15
	Iso-enhancement	11	29		
	Hypo-enhancement	4	19		
Homogeneity	Homogeneous	48	58	21.05	*<*0.01
	Heterogeneous	2	46		
Wash in	Fast	31	57	0.71	0.40
	Synchronous	14	33	0.22	0.64
	Slow	5	14	0.37	0.54
Wash out	Fast	17	50	2.72	0.10
	Synchronous	8	31	3.40	0.70
	Slow	25	23	12.24	0.00

### The independent indicators correlated with RCCs

**Figure 3:**
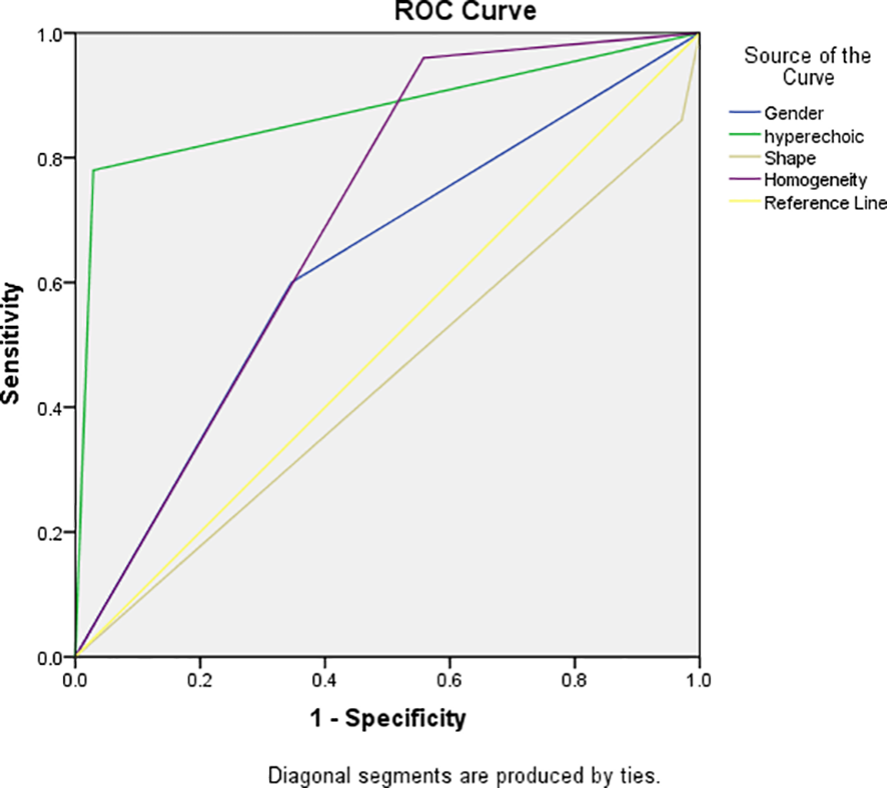
Receiver operating characteristic (ROC) curve demonstrated sensitivities and specificities of significant indicators of fat-poor AML. The areas under the curves were 0.88 and 0.70 for hyperechoic and enhancement uniformity, respectively.

A multifactorial analysis was conducted to discern independent indicators associated with fat-poor AML. The findings indicate that gender, morphology, internal hyperechoic features, and enhancement uniformity are autonomous factors correlated with fat-poor AMLs (**Table 4**). Notably, internal hyperechoic features, characterized by starry sky and crescent moon signs, along with enhancement uniformity, demonstrated the most robust correlations. The area under the ROC curve for internal hyperechoic features was 0.88 (95% CI: 0.80–0.95), featuring a sensitivity of 78.00%, specificity of 97.12%, positive predictive value of 92.85%, and negative predictive value of 90.18%. Conversely, the ROC curve area for enhancement uniformity was 0.70 (95% CI: 0.62–0.78), with a sensitivity of 96.00%, specificity of 44.23%, positive predictive value of 45.28%, and negative predictive value of 95.83% (**Table 5**, **Figure 3**). The diagnostic performance of fat-poor AMLs of varying sizes with respect to hyperechoic and homogeneity is detailed in **Table 6**.

**Table 4:** Multivariate analysis with variable selection for predicting fat-poor AMLs.

Characteristics	B	SE	OR (95%CIs)	*P*-value
Gender	-1.94	0.79	0.14	0.01
Age	0.00	0.02	1.00	0.98
Shape	3.35	1.15	28.52	*<*0.01
hyperechoic	-5.89	1.18	0.003	*<*0.01
Homogeneity	-4.11	1.35	0.02	*<*0.01
Wash out	0.14	0.71	1.15	0.84

**Table 5:** ROC analyses of the independent variables for predicting fat-poor AMLs.

Variables	Cut-off value	Sensitivity (%)	Specificity (%)	positive predictive value (%)	negative predictive value (%)	AUC (95%CIs)
Gender	Female	60.00	65.38	45.45	77.27	0.63
Shape	Irregular	14.00	97.12	70.00	97.12	0.44
Hyperechoic	exist	78.00	97.12	92.86	90.18	0.88
Homogeneity	Homogeneous	96.00	44.23	45.28	95.83	0.70

**Table 6:** The performance of hyperechoic and Homogeneity based on the size of fat-poor AMLs.

Characteristics	Sensitivity (%)	Specificity (%)	positive predictive value (%)	negative predictive value (%)	Accuracy(%)
≤5cm (n=154)					
Hyperechoic	78.00	97.12	92.86	90.18	90.91
Homogeneity	96.00	44.23	45.28	95.83	61.04
≤3cm (n=96)					
Hyperechoic	75.00	96.88	92.31	88.57	89.58
Homogeneity	77.78	97.44	42,47	90.48	55.21
≤2cm (n=57)					
Hyperechoic	96.88	34.38	93.33	95.65	91.23
Homogeneity	100.00	28.20	39.13	100.00	50.88

## Discussion

Renal AML can be classified into two subtypes based on the proportion of fat within the tumor: fat-poor AML and classic AML (1). The diagnosis of most renal AML relies on the identification of visible fat on CT or MRI (3). Although specific imaging features can be helpful for diagnosing fat-poor AML, such as homogeneous enhancement on contrast-enhanced CT (CECT) (21), and high T1 signal, low T2 signal, and delayed enhancement on MRI (22), their use is limited by concerns about nephrotoxicity, cost, and the need for contrast material administration (23). CEUS combined with CUS has limited application in the differential diagnosis of renal tumors (24–26). We investigated patient demographic characteristics that might aid in distinguishing fat-poor AML from RCC. We found a significantly higher prevalence of female patients with renal AML compared to males (*p*<0.01). In our study, 60.0% of patients with fat-poor AML were female, whereas only 34.6% of patients with RCC were female, which is consistent with previous reports (27). The mean age in the RCC group was 53.46 ± 15.43 years, compared to 48.54 ± 12.71 years in the fat-poor AML group. This difference in age was statistically significant (*p*=0.04). The distribution of tumors across the right, left, upper, middle, and lower kidneys did not reveal any statistically significant differences.

**Figure 4:**
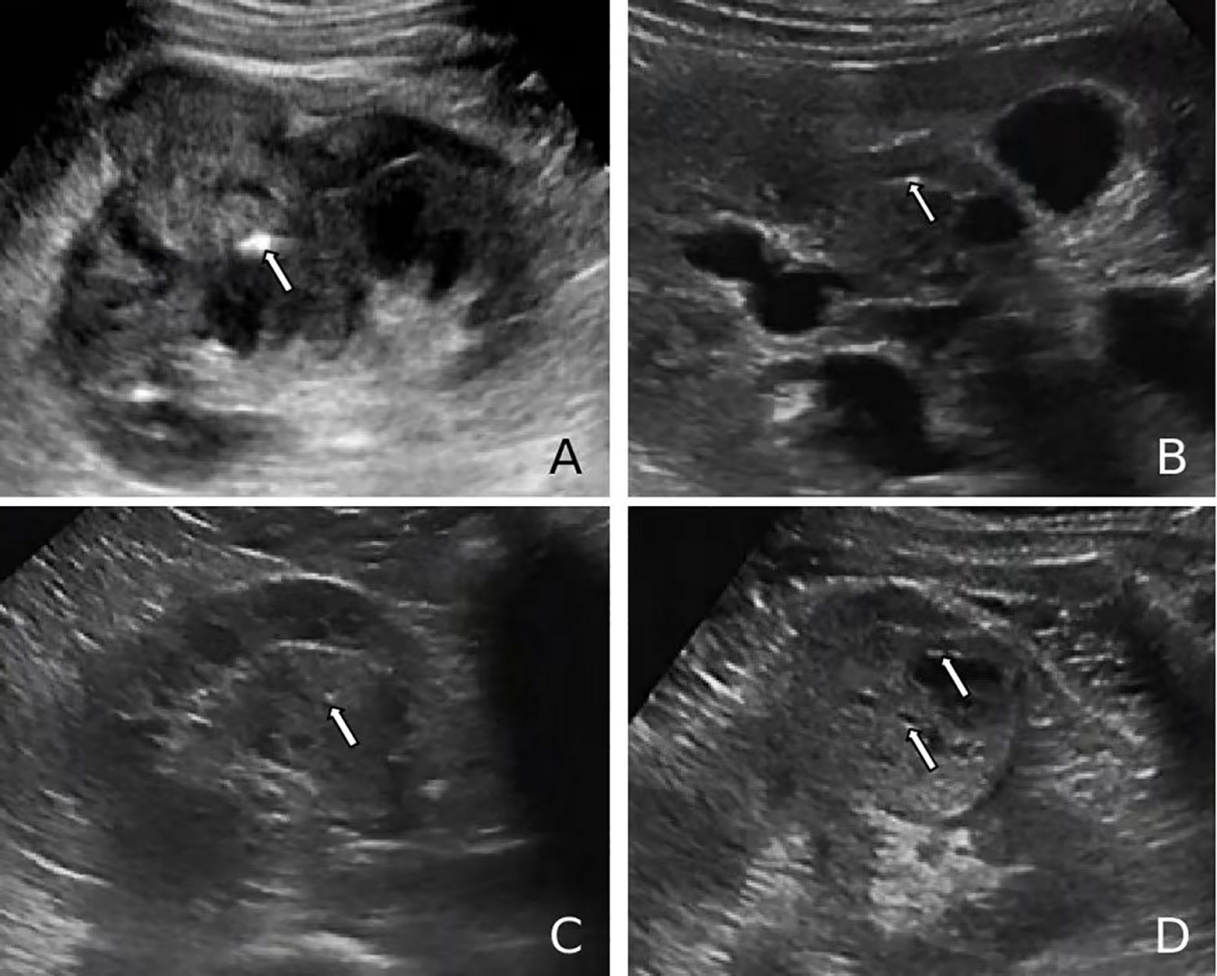
**(A, B)** Ultrasound images of renal cell carcinoma (RCC) demonstrating a markedly hyperechoic intratumoral lesion (arrows), exceeding the echogenicity of the renal sinus, consistent with a calcification focus. **(C, D)** Ultrasound images of RCC showing punctate hyperechoic foci within the lesion and anechoic areas peripherally (arrows), suggestive of posterior acoustic enhancement and calcified cyst wall.

Hypoechoic renal masses are often considered malignant on ultrasound. Conversely, hyperechoic and isoechoic masses are typically considered benign (28). However, the distinction in internal echo patterns between fat-poor AML and RCC remains challenging due to the lack of fat in some AMLs, leading to a predominantly hypoechoic appearance on ultrasound. Our study identified the presence of internal hyperechogenicity within hypoechoic renal masses as an independent predictor of fat-poor AML (*p*=0.00, OR=0.01, AUC=0.88). The sensitivity, specificity, positive predictive value, and negative predictive value were 78.00%, 97.12%, 92.85%, and 90.18%, respectively. When the size is ≤2 cm, the sensitivity for hyperechoicity increases, but the specificity decreases. Importantly, the presence of residual fat within fat-poor AMLs manifests as internal hyperechogenicity on ultrasound, potentially aiding in differentiation from RCC. Fat in well-organized areas appears hypoechoic, while fat within a disorganized mass appears hyperechoic, possibly due to the presence of reflective interfaces. Preoperative ultrasound findings and postoperative pathology confirmed that internal hyperechogenicity within hypoechoic renal masses represented fat tissue. The distribution of this hyperechogenicity also demonstrated a pattern. The predominant cellular components of AML are smooth muscle or vascular perithelial-like cells. Scattered residual fat tissue manifested as a “starry sky” sign on ultrasound (**Figure 1**), indicating minimal fat content. When compressed to one side, the residual fat can appear as a crescent sign, characterized by localized arc-shaped hyperechogenicity at the tumor margin (**Figure 2**). Notably, this finding was rarely observed in RCC (3/104, 2.88%), with limited reports in the literature. The renal sinus, rich in fat content, served as a reference for interpreting internal hyperechogenicity within the masses. While RCC typically displays slightly hyperechoic internal features, it may also exhibit linear or granular high-to-strong hyperechogenicity. However, image magnification revealed that linear hyperechogenicity in RCC stemmed from posterior echo enhancement of tiny cysts (**Figure 4**). Granular hyperechogenicity often presented with a comet tail or acoustic shadow, and these postoperative pathological findings confirmed the presence of calcifications. The frequent occurrence of hemorrhage, necrosis, and cystic degeneration in RCC contributes to these findings (29), highlighting that these hyperechoic areas do not represent fat tissue. In conclusion, the presence of hyperechoic areas within hypoechoic renal masses is a characteristic feature of fat-poor AML, which can significantly improve diagnostic accuracy. Patients exhibiting these features may undergo conservative treatment rather than surgical intervention, thereby avoiding unnecessary surgery and benefiting the patient. However, the presence of hyperechoic areas combined with significant indicators in CUS and CUES does not further enhance the diagnostic efficacy for fat-poor AML.

**Figure 5:**
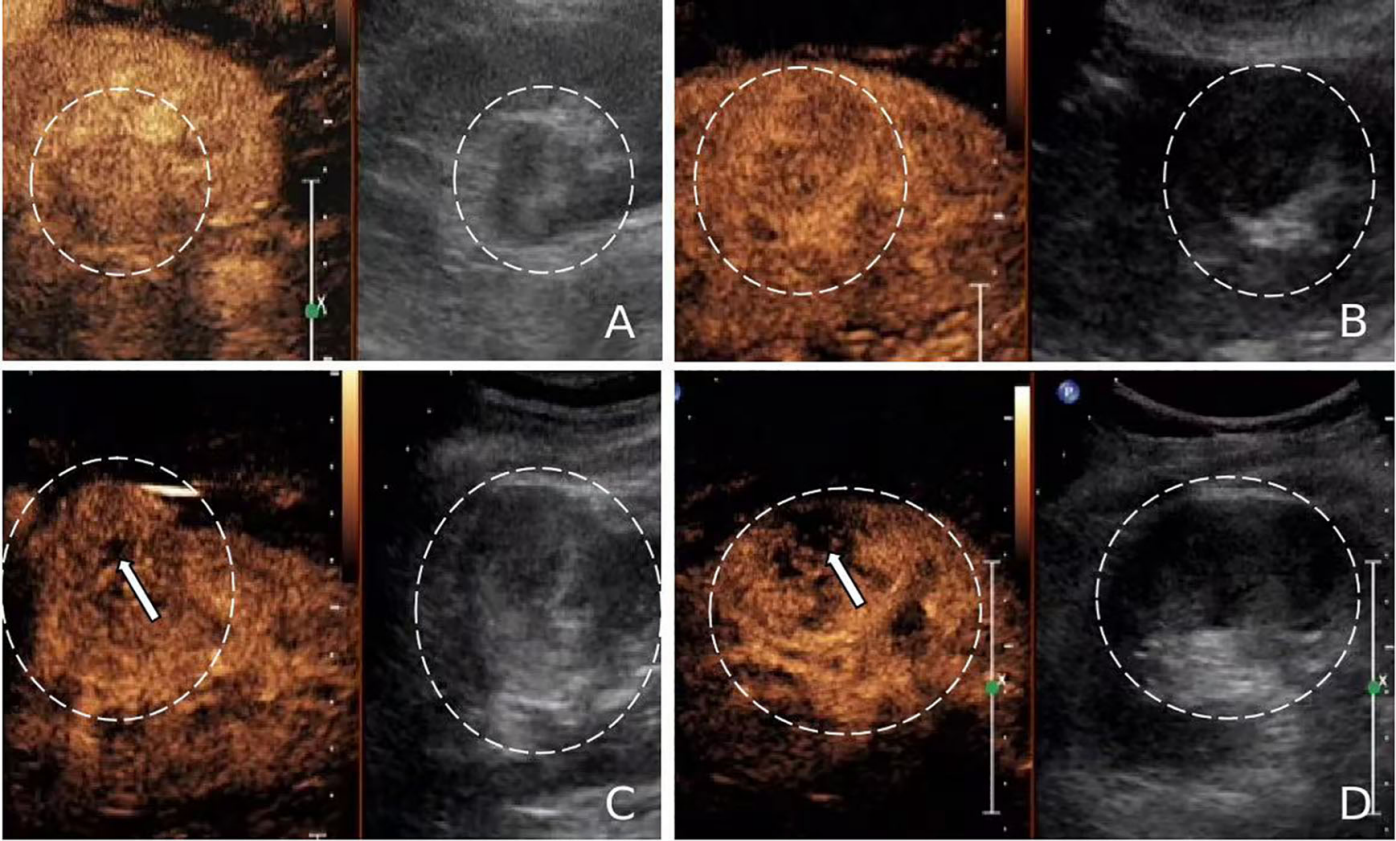
**(A, B)** Contrast-enhanced ultrasound (CEUS) images of fat-poor angiomyolipoma(AML) (circled) showing uniform enhancement without unenhanced areas. **(C, D)** CEUS images of renal cell carcinoma (RCC) (circled) exhibiting heterogeneous enhancement with unenhanced areas (arrows).

Several studies support the utility of CEUS in differentiating between fat-poor AML and RCC (30–34). Our analysis identified uniform enhancement on CEUS as a statistically significant factor for distinguishing these entities in both univariate and multivariate models (*p<*0.01). Consistent with this, 48 of 50 (96.0%) fat-poor AMLs in our study demonstrated uniform enhancement. This observation likely reflects the slow growth pattern of smaller AMLs, which reduces the likelihood of encountering necrosis, cystic degeneration, or hemorrhage. Hemorrhage, if present, is typically observed in larger AMLs and manifests as spontaneous bleeding (35). Our findings are further corroborated by Hongli Cao et al (28), who reported uniform enhancement in 77.3% (17/22) of AMLs on CEUS, suggesting a higher prevalence of uniform enhancement in AML compared to RCC. Conversely, the rapid growth of RCC tumor cells and their susceptibility to ischemic necrosis contribute to the more frequent occurrence of non-uniform enhancement on CEUS in this malignancy (36)(**Figure 5**). Our study demonstrated delayed contrast enhancement in half of the fat-poor AML cases. However, multivariate analysis did not identify delayed enhancement as an independent predictor for differentiating AML from RCC. This finding contrasts with the work of Liu et al (37), who reported persistent enhancement in 88.0% (29/33) of AMLs. This discrepancy may be attributed to the composition of the AMLs studied. In their study, 88.0% were classic type, rich in fat content. Conversely, all AMLs in our investigation were devoid of fat. These observations suggest that intra-lesional fat content might influence the pattern of contrast agent washout on CEUS. Further studies are warranted to elucidate this potential association.

Our study identified intriguing morphological differences between fat-poor AML and RCC on multivariate analysis (*p<*0.01). Although the area under the receiver operating characteristic curve (AUC) was low (0.44), shape emerged as an independent predictor in the model. Fat-poor AMLs frequently exhibited irregular morphology, with a predominance of mushroom-like shapes, particularly in lesions near the renal capsule. This finding aligns with the morphology of classic AMLs (38). The absence of a restraining capsule in fat-poor AMLs likely allows for expansive growth patterns. When subjected to uneven mechanical forces, these lesions are more prone to adopting irregular shapes. Conversely, RCC, being a malignant tumor, exhibits expansive growth with the formation of a pseudocapsule that interacts with surrounding renal parenchyma, resulting in a more regular morphology (39). Several studies have described unique radiological features of fat-poor AML, including the corner interface sign, ice cream cone sign, and spilled beer sign (40). Marshall Strother et al (41), further identified the spilled beer sign (OBS) as a potential marker for improved diagnostic sensitivity of hypoechoic AML.

Our study has several limitations: First, this is a single-center retrospective study, and the number of pathological specimens obtained from fat-poor AMLs is limited. A study by Fank et al (42). showed that the frequency of benign tumors among resected renal tumors ≤5 cm is 9.9%, and the incidence of hypoechoic types in RCC is relatively low. To address this, we collected data over a 10-year span, during which ultrasound techniques and imaging have evolved. These factors may introduce biases, necessitating multicenter, large-scale prospective studies to validate our findings. Second, our analysis was limited to fat-poor AMLs and RCCs, without considering their subtypes, other benign tumors, or other malignant tumors; future studies could include a broader range of lesions. Third, the assessment of hyperechoic areas within tumors was based on subjective human judgment, which is prone to error. Combining this with CEUS might aid in diagnosis, as hyperechoic areas in fat-poor AML show uniform enhancement, while calcifications and post-cystic echo enhancement exhibit non-uniform enhancement under CEUS. The use of artificial intelligence for image analysis could provide more objective and accurate results. Fourth, the cases we selected were based solely on ultrasound findings, without incorporating imaging diagnostic criteria from CT or MRI, which introduces significant subjectivity. This can be addressed in future studies by incorporating stricter imaging diagnostic criteria, or by establishing a multidisciplinary evaluation team to minimize subjective factors in surgical decision-making, thereby reducing unnecessary resections of benign tumors.

Overall, we hope to conduct long-term follow-up multicenter prospective studies in the future, incorporating a broader range of tumor types and imaging modalities, to further validate the diagnostic performance of CUS and CEUS in fat-poor AMLs, thereby reducing the resection rates of benign tumors.

## Conclusion

Our study suggests that intralesional hyperechogenicity, manifested as starry sky or crescent moon signs on ultrasound, and uniform contrast enhancement may function as independent predictors of fat-poor AML. This imaging signature could potentially aid in differentiating fat-poor AML from RCC, particularly in lesions less than 5 cm. This distinction might facilitate more targeted clinical management strategies, potentially reducing unnecessary surgical interventions.

## Data Availability

The datasets presented in this study can be found in online repositories. The names of the repository/repositories and accession number(s) can be found in the article/supplementary material.
